# Bridging racial and ethnic disparities in cancer research

**DOI:** 10.1002/cnr2.1871

**Published:** 2023-08-01

**Authors:** Richa Sharma, Amit K. Tiwari

**Affiliations:** ^1^ John Wiley & Sons Noida India; ^2^ Department of Pharmaceutical Sciences, College of Pharmacy University of Arkansas for Medical Sciences Little Rock Arkansas USA

The issue highlights the need to bridge racial and ethnic disparities in cancer research to promote equitable healthcare outcomes. Our knowledge of unique risk factors and disease progression patterns in minority groups is hindered by underrepresentation of these groups in clinical trials and genomic databases. Thus, personalized treatment strategies cannot be developed effectively. Biological diversity among racial and ethnic groups adds further complexity, requiring more nuanced, population‐specific research. Additionally, socioeconomic factors, language barriers, and cultural differences also contribute to a complex tapestry of disparities that impede equal access to healthcare. There is also a longstanding mistrust in healthcare systems, rooted in historical injustices, which discourages minority participation in research and clinical trials. Additionally, the lack of universally accepted racial and ethnic classifications hinders comprehensive comparative studies. In order to ensure that all communities, regardless of their racial and ethnic backgrounds, benefit from advances in cancer research, it is crucial to overcome the challenges. As a result, we will be able to foster healthcare equity as well as gain a deeper understanding of cancer's complexities. This special issue focuses on racial and ethnic disparities across oncology, with a focus on breast, prostate, lung, and liver cancer.

Breast cancer (BC) impacts diverse socioeconomic and ethnic groups. In a study by Zhang et al., the global age‐standardized incidence rate of BC increased by 14.31% from 1990 to 2019, while mortality rates declined. It is found that incidence rates correlate with sociodemographic factors, such as alcohol consumption in high‐income regions, such as Europe, and high levels of fasting plasma glucose in Latin America and Africa. Through 2044, future projections predict a rapid rise in incidence, particularly among women aged 35–60.^1^ Additionally, Sassi et al. found that Tunisian and Algerian women with BC had significant differences in their clinical and histopathological characteristics compared with Western women. They were younger at diagnosis, had larger tumors, and had more axillary lymph nodes involved. A tailored approach in medical intervention is required to address the stark differences in cancer profiles and outcomes across regions.^2^ The comprehensive review by Zajac et al. uncovers disparities within tumor microenvironments, especially in African American women with triple‐negative breast cancer (TNBC). It highlights that the elevated levels of hormones like resistin, pro‐inflammatory cytokines like IL‐6, and chemokines like CCL2 and CXCL12 stimulate the recruitment of macrophages like M2, vascular endothelial growth factors, and regulatory T lymphocytes, which promote tumor growth and metastasis. These disparities, coupled with socioeconomic barriers, prevent access to advanced therapies. A precision oncology approach, considering the unique biological and socioeconomic characteristics of individual patients, is necessary to address these disparities.^3^

There are alarming racial disparities in prostate cancer, especially between African American Men (AAM) and European American Men (EAM), with the former often experiencing worse outcomes. These disparities are illuminated by two critical studies. Among black African patients from the sub‐Saharan region, Mougola Bissiengou et al. conducted a groundbreaking study on the tumor microenvironment of prostate cancer and benign prostatic hyperplasia. They reported that CD73, a T‐regulatory surface enzyme, was highly expressed in epithelial‐stromal cells and that CD8 T lymphocytes and natural killer cells were selectively infiltrated. There was seven times more CD73 expression in prostate cancer stromal tissues than in BPH tissues, and prostate cancer patients had higher PSA concentrations, indicating CD73 as an immunotherapy target.^4^ According to Le et al., despite the higher prostate cancer risk of AAM, they are underrepresented in genetic studies and clinical trials. As a result of this underrepresentation, genomic predictor models and insights from genome‐wide association studies (GWAS) may not be as applicable to AAMs. The lack of understanding of biological characteristics and differences between AAM and EAM could impede personalized treatment. Accordingly, Le et al. proposed that bridging the divide and achieving health equity requires increased recruitment and engagement of AAMs.^5^

Cancer‐related deaths caused by hepatocellular carcinoma (HCC) are also affected by both genetic and nongenetic factors. Mutations in genes such as TP53, CTNNB1, ARIC1A, and CDKN2A are among the genetic factors, while nongenetic factors include alcohol consumption, aflatoxin exposure, age, gender, and diseases such as nonalcoholic fatty liver disease, hepatitis B, and hepatitis C. HCC, which disproportionately affects men and minorities, differs by geographical location, with patients from non‐Caucasian backgrounds experiencing poorer survival outcomes. Chavda et al. present an epidemiological perspective on factors influencing deviations from conventional treatment for HCC. Chavda et al. emphasize the importance of having consistent, evidence‐based assessment and treatment protocols, supported by international clinical guidelines, to improve the treatment outcome of HCC. Global collaborations between researchers, healthcare providers, and advocacy groups can bring about advancements in liver cancer research through such standardized approaches.^6^

The review by Pathak et al. examines the current clinical literature on the disposition, effectiveness, toxicity, and possible effects of chemotherapeutic drugs on ethnic groups. In an effort to address how genetic and nongenetic factors influence anticancer treatment responses, they expanded the concept of pharmacoethnicity, a core aspect of pharmacogenomics, the study of how genes from different populations affect drug processing and effects. In some cases, these variants are the result of polymorphisms in metabolism‐related genes, which affect the pharmacokinetics and pharmacodynamics of the drug. Understanding the impact of pharmacoethnicity on chemotherapy can lead to more effective and tolerated treatments. Pathak et al. also suggested diversifying the study population, addressing implicit biases among providers, and ensuring personalized treatment to reduce these disparities.^7^

Bharmjeet and Das, in their review, highlighted the inequities across different ethnic and economic groups. These inequities exist at multiple stages of diagnosis, prognosis, treatment, and access to healthcare facilities. The impact of industrialization on diet, lifestyle, and socioeconomic conditions has contributed to disparities in cancer incidence and mortality worldwide, despite advancements in cancer care and treatment. There is still an unequal distribution of medical facilities and trained staff, inequalities in screening, and prohibitive treatment costs.^8^ Diverse population groups/races/ethnicities need to be represented in clinical trials. In another report, Aslam et al. determined the trends in race and sex reporting in lung cancer phase III clinical trials published over the last 35 years. Aslam and colleagues analyzed that female participation has improved with time at a rate of 0.65% per year and the trends over time are promising; nonetheless, African Americans are still underrepresented, and their rate of participation decreased at a rate of −0.30% per year despite the rising incidence in lung cancer. However, this decrease in participation was accompanied by an increase in the rate of Asian participants by a rate of 1.07% per year with no change in rates of participation among Hispanics. Thus, the study substantiates the evidence that continues to highlight the disparities surrounding races in clinical trials.^9^ In addition, to enhance cancer treatment outcomes, Pathak and colleagues and Bharmjeet and Das emphasize the importance of collaboration among researchers, healthcare providers, and policymakers.^7^, ^8^

In summary, achieving equitable progress in cancer care and mortality reduction requires addressing global disparities, including socioeconomic status, education, diagnostic methods, treatment methods, clinical trial participation, and immunotherapy and personalized treatment. In order to reduce racial and ethnic disparities in cancer outcomes, we will need a multidisciplinary approach: improving the collection of health data, genetics, and the sociocultural environment; developing targeted treatments based on genomic datasets; promoting early detection through screenings; and reducing social taboos through education. Health equity programs, comprehensive insurance policies, and universal standard treatments are crucial to eliminating cancer disparities.
